# Real time and delayed effects of subcortical low intensity focused ultrasound

**DOI:** 10.1038/s41598-021-85504-y

**Published:** 2021-03-17

**Authors:** Joshua A. Cain, Shakthi Visagan, Micah A. Johnson, Julia Crone, Robin Blades, Norman M. Spivak, David W. Shattuck, Martin M. Monti

**Affiliations:** 1 Department of Psychology, University of California Los Angeles, Pritzker Hall, Los Angeles, CA 90095 USA; 2 Department of Neurology, University of California Los Angeles, Los Angeles, 90095 USA; 3 Department of Neurosurgery, University of California Los Angeles, Los Angeles, 90095 USA; 4 Department of Psychiatry, University of California Los Angeles, Los Angeles, 90095 USA; 5 Brain Injury Research Center (BIRC), Department of Neurosurgery, University of California, Los Angeles, CA 90095 USA

**Keywords:** Cognitive neuroscience, Biophysics, Neural circuits

## Abstract

Deep brain nuclei are integral components of large-scale circuits mediating important cognitive and sensorimotor functions. However, because they fall outside the domain of conventional non-invasive neuromodulatory techniques, their study has been primarily based on neuropsychological models, limiting the ability to fully characterize their role and to develop interventions in cases where they are damaged. To address this gap, we used the emerging technology of non-invasive low-intensity focused ultrasound (LIFU) to directly modulate left lateralized basal ganglia structures in healthy volunteers. During sonication, we observed local and distal decreases in blood oxygenation level dependent (BOLD) signal in the targeted left globus pallidus (GP) and in large-scale cortical networks. We also observed a generalized decrease in relative perfusion throughout the cerebrum following sonication. These results show, for the first time using functional MRI data, the ability to modulate deep-brain nuclei using LIFU while measuring its local and global consequences, opening the door for future applications of subcortical LIFU.

## Introduction

While routinely used to modulate the cortex non-invasively, established neuromodulatory techniques such as transcranial magnetic stimulation (TMS) and transcranial electrical stimulation (tES) remain limited in both the domains of spatial precision and the depth of their influence^1,2^. Because such non-invasive protocols are unable to selectively target structures below the cortical mantle, reversible neuromodulation of the deep brain has been limited to invasive techniques, such as deep brain stimulation (DBS), which involve the surgical implantation of electrodes and are thus limited to (severe) patient populations. However, the emerging technology of low-intensity focused ultrasound (LIFU) has increasingly been shown to address this gap; indeed, LIFU has demonstrated the capacity to both inhibit as well as excite subcortical tissues safely and reversibly in model organisms (i.e., rats^3–7^, pigs^8^, and macaques^9^, as well as, recently, healthy human subjects^10^) with a spatial precision far exceeding that of TMS or tES^1,2,8,11,12^.

The ability of LIFU to selectively modulate subcortical tissue non-invasively potentially makes way for causal inferences in the study of subcortical networks as well as the treatment of many neurological conditions. The lentiform nuclei are of particular interest for their role in a cortico-basal ganglia-cortical circuit ostensibly mediating motor refinement, cognitive functioning, and arousal^13,14^. Moreover, these structures are relatively accessible to LIFU through the temporal window, the thinnest section of the temporal bone and, thus, the ideal cranial entry-point for minimizing ultrasound attenuation and refraction through skull when utilizing a single-element transducer. Despite the centrality of basal ganglia-cortical circuits to several aspects of human cognition^13^, knowledge of their precise structure and function remains incomplete and evolving. Only recently, for instance, has the field begun to appreciate the contribution of the lentiform nuclei to maintaining electro-cortical and/or behavioral arousal^15,16^, as evidenced by both animal models and human studies, putatively through a recently identified extra-thalamic direct pallido-cortical pathway^17–19^. LIFU thus promises the unprecedented ability of performing causal investigation into the role of these circuits in healthy volunteers. While clinical translation of this technique is already ongoing^20^ in the context of Disorders of Consciousness^14^ (DOC) after severe brain injury, pallidal LIFU is likely to be applicable to other conditions such as obsessive–compulsive disorder^21^, Tourette syndrome^22^, treatment-resistant depression^23^, Huntington’s disease, and Parkinson’s disease^24,25^.

**Figure 1:**
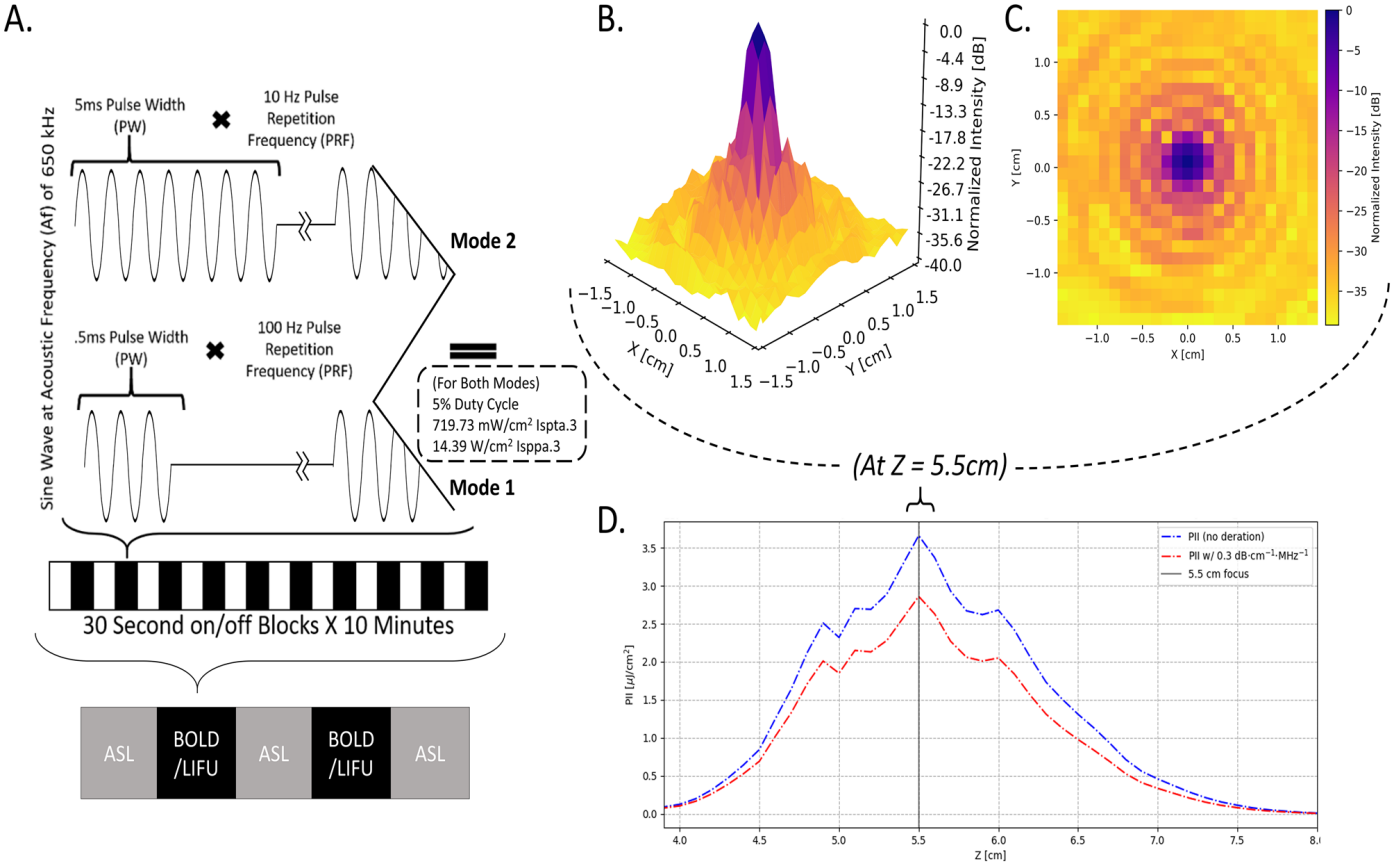
Sonication Parameters, Experimental Design, and Water Tank Measurements of Beam Properties. (**A**) Parameters for each LIFU mode utilized including pulsing schedule, block design, and intensity. I_sppa.3_ = Spatial Peak Pulse Average Intensity. I_spta.3_ = Spatial Peak Temporal Average Intensity; “.3” denotes deration (attenuated intensity at 0.3 dB/cm-MHz) through human tissue. Here, we have applied LIFU in two sessions, utilizing different parameter sets in each with LIFU Mode 1 having a pulse repetition frequency (PRF) of 100 Hz PRF and a pulse width (PW) of 0.5 ms PW while LIFU Mode 2 = 10 Hz PRF, 5 ms PW; all other factors including duty cycle (DC) = 5% and intensity I_spta.3_ (Spatial Peak Temporal Average)^42^ = 720 mW/cm^2^ ; I_sppa.3_ (Spatial Peak Pulse Average)^42^ = 14.40 W/cm^2^ were held constant. (**B**,**C**) Intensity in the radial plane (X/Y plane, extending from focal point of ultrasound beam 5.5 cm from transducer surface) shown in both 3 (**B**) and 2 (**C**) dimensions. A 50% (− 3 dB) reduction in peak intensity occurs in an area approximately 0.5 cm in width. Note that the decibel scale is nonlinear and -3 dB approximately corresponds to a 50% reduction in intensity; this scale is normalized to maximal intensity, where peak intensity equals 0 dB. (**D**) Intensity in the longitudinal plane (Z plane, extending from transducer) in absolute (pulse intensity integral (PII); “.3”denoting absorption in human tissue at 0.3 dB/cm-MHz) values of Z correspond to distance from the transducer surface. Note the peak intensity 5.5 cm from the transducer surface and that a 50% (− 3 dB) reduction in peak intensity is found in an area approximately 1.5 cm in length.

Here, we administered two sessions of deep-brain LIFU in 16 healthy volunteers. In each session, participants received two 5-min doses (i.e., runs) of LIFU aimed at the left globus pallidus (GP). Each run featured, in an off/on block design, ten 30-s blocks of sonication each preceded by 30-s of rest (see Fig. 1A). Given the current uncertainty with respect to how different sonication parameters (e.g., duty cycle, pulse repetition frequency, pulse width) relate to local and distal brain modulation, two sonication modes modeled after prior work^4–7^ were administered, one per session. To assess the “online” (concurrent effects) effects of LIFU, we collected T2*-weighted blood oxygenation level dependent (BOLD) data during each sonication run. To assess “offline” (longer-lasting) effects of LIFU, we also collected perfusion-weighted arterial spin labeling (ASL) data prior to the first sonication run, in-between the two runs, and after the second run. The main aims of the work were to assess the online and offline topography (local and global) and valence (i.e., up-/down-modulation) of the brain response to each sonication setting.

## Results

We present the results of this work in three main sections. First, we describe our sonication settings as well as the spatial characteristics of our ultrasound beam when passed through free water (measured empirically) and bone (simulated using k-wave^26^ in Matlab). Second, we report online local and global effects of LIFU sonication with an ROI analysis of the principal target (left GP) and proximal structures (i.e., left putamen and left thalamus; right putamen, thalamus, GP as controls), as well as a full brain analysis of the same BOLD data. Finally, we describe offline effects of LIFU both locally, with an ROI analysis of ASL perfusion data, and globally, with a full-brain analysis of the same perfusion data.

## Ultrasound waveform through water and bone

**Figure 2:**
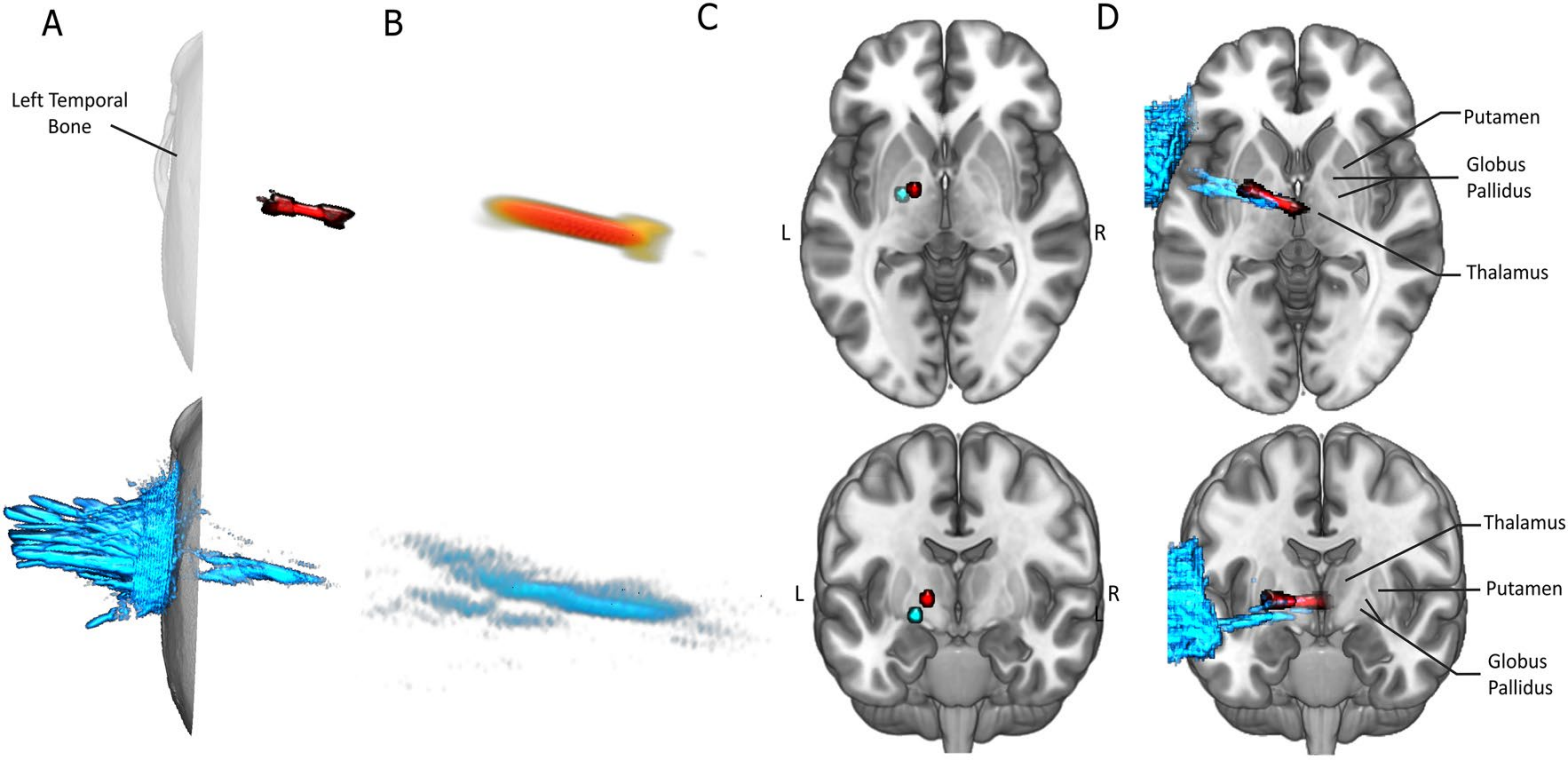
Numerical Modeling through Bone and Water. Here, one 5 ms pulse (corresponding to 1 pulse of Mode 2) of our ultrasound beam is simulated twice, in water (red), and through the temporal bone of a human computed tomography (CT) image (cyan). The same trajectory, which targets the left GP, was used in both simulations. The maximal pressure for each voxel over the course of the simulation is visualized. Only Voxels exceeding 50% (-3 dB) of the maximum in-brain pressure are presented. (**A**) Depiction of the effect of bone on beam shape and position. Note that bone (bottom, cyan) appears to flatten, deform, and laterally retract the ultrasound beam compared to the water condition (top, red). However, the general expression of an elongated beam and its general location is retained. Further note that most energy (**A**, bottom) is deposited into and reflected off bone when it is present. (**B**) Higher-resolution depiction of ultrasound beam in water (B, top) and through bone (B, bottom). (**C**) Location of maximum pressures following propagation through water (red) and through bone (cyan). Points were mapped into MNI space for visualization. Note that both reside inside the left pallidal target. While the effect of bone moves the peak pressure somewhat ventral and lateral, the total translation is 0.93 cm. (**D**) Depiction of whole ultrasound beam through water (cyan) and bone (red). Simulations were mapped into MNI space for visualization. Note that energy deposition into portions of the left lentiform nuclei and left thalamus exceeded the threshold of − 50% maximum pressure.

Previous simulations of ultrasound propagation have demonstrated the general maintenance of focal shape and focal location when passing through the human skull, including through the temporal bone^27^. To ensure that these results generalize to our LIFU parameters (see Fig. 1) and our trajectory, we have simulated, in three dimensions, 5 ms (equivalent to 1 pulse in LIFU Mode 2) of LIFU propagation at our parameters through the temporal bone of a high-resolution CT (Visible Human^28^) and into the left GP. Regarding refraction, the point of maximum pressure of this simulation, compared to simulation through water alone, deviated by 0.93 cm, in the range of previous findings^27,29^. As depicted in Fig. 2C,D, our results suggest that energy deposition remains inside the targeted left GP while not significantly impacting ventral or dorsal structures (e.g., left hippocampus), despite some expected deviation. The thresholded (− 50%) pattern of energy deposition subsumes portions of the left thalamus and left putamen, supporting the attention given here to these structures (Fig. 2D, Cyan). Regarding attenuation, skull bone attenuated the energy reaching the brain significantly, as expected, resulting in a peak in-brain pressure 12.35% of that simulated in water alone. It is important to note that the depictions of our ultrasound beam here, for both water and bone simulations, are thresholded at an arbitrary value: (− 50%) from maximal in-brain pressure. While the skull deforms and “flattens” the energy deposition to some extent, resulting in a larger area exceeding that − 50% threshold (see Fig. 2B), an oblong focus of high intensity remains with a degree of refraction (0.93 cm) that retains energy deposition into pallidal tissues. Note that cumulative energy deposition over time was recorded at the point of max intensity and is highly linear (see Figure S5), suggesting that these results also generalize to the shorter 0.5 ms pulse.

## Region of interest (ROI) analysis

### BOLD—ROI

In this study, each participant underwent two sonication sessions: one with the “Mode 1” parameter set and one with the “Mode 2” parameter set. In each session, participants underwent two sonications (i.e., “runs”, with the same parameters) delivered in a simple block design featuring ten repetitions of 30 s of baseline followed by 30 s of sonication. Each participant thus underwent a total of 4 sonications: two with Mode 1 LIFU, in one session (order randomized across participants), and two with Mode 2 LIFU, in the other session. Firstly, we assessed the local influence of LIFU during sonication using both parameter sets on BOLD signal as compared to baseline (30 s inter-sonication blocks when no ultrasound is applied) by extracting the effect of LIFU within 6 ROI’s—the Putamen, the Globus Pallidus, and the Thalamus from each hemisphere. Given that our LIFU application was likely to impact only the left lateralized structures, as confirmed by numerical modelling (see Fig. 2), the right lateralized structures act as control regions in this analysis. Thus, a 2 × 2 × 2 × 3 ANOVA (as well as its Bayesian equivalent) was performed with Hemisphere (Left, Right) Parameter Set (Mode 1, Mode 2), Run (Run 1, Run 2), and ROI (Left Putamen, Left GP, Left Thalamus) as factors. While no main effects were observed, a significant Hemisphere by Run by ROI by Parameter Set interaction compelled follow-up three-way ANOVAs for each hemisphere. As expected, no significant effects were found in the right hemisphere structures.

However, the 2 × 2 × 3 repeated measures ANOVA (as well as its Bayesian equivalent) performed on left lateralized structures with parameter set (Mode 1, Mode 2), Run (run 1, run 2 within session), and ROI (Left Putamen, Left GP, Left Thalamus) as factors revealed a significant effect of parameter set, *F*(1,15) = 5.442 , *p* = 0.034, BF_Inclusion_ = 78.620. A significant interaction (in the frequentist but not Bayesian approach) between parameter set, Run, and ROI was also found *F*(1,15) = 9.055, *p* = 8.36e^−4^, BF_Inclusion_ = 0.647). Based on this finding as well as our a-priori expectation of regional effects from LIFU (see Discussion), a follow-up two-way ANOVA was performed for each ROI. While no significant effects were found for the Left Putamen, a significant effect of parameter set was found in both the Left GP (*F*(1,15) = 4.585, *p* = 0.049, BF_inclusion_ = 2.49) as well as the Left Thalamus *F*(1,15) = 5.115, *p* = 0.039, BF_inclusion_ = 1.53) with reduced BOLD found during sonication in LIFU Mode 1 as compared to LIFU Mode 2 for both ROIs. To assess if either parameter set induced a change in BOLD signal from baseline, marginal means were assessed for each parameter set for each ROI. A reduction in BOLD from baseline during sonication in Mode 1 was found in the Left GP, *t*(15) = -2.923 , *p*_*Šidák*_ = 0.013, BF_10_ = 5.54, and the Left Thalamus, *t*(15) = -2.436 , *p*_*Šidák*_ = 0.042, BF_10_ = 3.62. These results suggest that sonication in Mode 1 significantly reduced BOLD signal in the Left GP and the adjacent Left Thalamus when compared to sonication in Mode 2 and when compared to baseline. Despite having the same acoustic intensity (i.e., I_spta_ = 720 mW/cm^2^) and duty cycle (i.e., 5%) as Mode 1, Mode 2 LIFU failed to induce any detectable effect on the BOLD signal in analyzed regions.

### ASL—ROI

**Figure 3:**
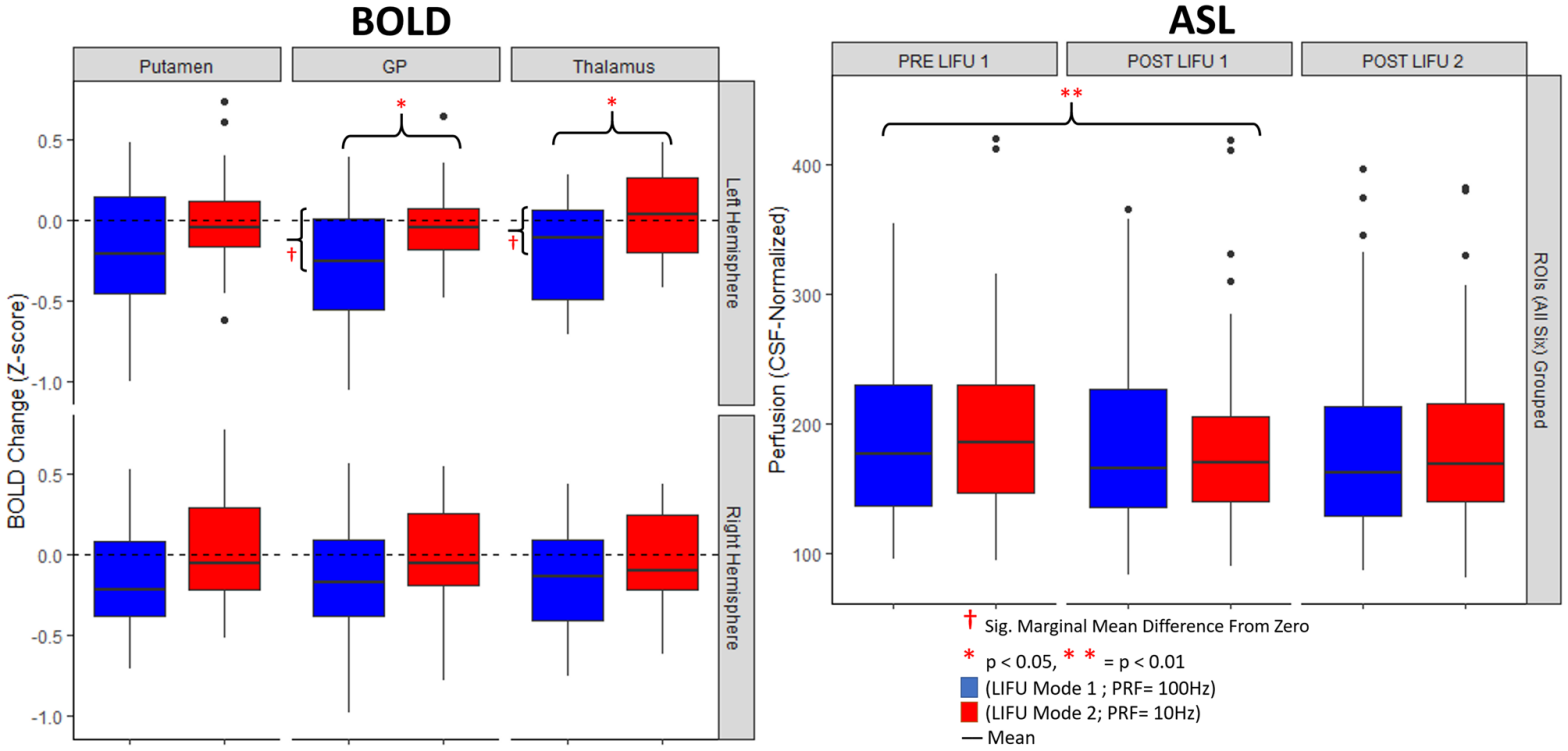
ROI analysis results. Left: online changes in BOLD signal during LIFU sonication blocks compared to inter-sonication blocks (i.e., baseline) for the target Left GP ROI, in the proximal Left putamen and Left thalamus ROIs, and in the right Putamen, GP, and Thalamus during Mode 1 (blue) and Mode 2 (red) sonication, compared to baseline (Red crosses indicate a significant difference from baseline for an individual condition while red Asterisks indicate significant difference across conditions). Right: offline changes in ROI perfusion (all six ROIs are grouped) before LIFU, after run 1, and after run 2 for each sonication mode (Red Asterisks indicate significant difference across conditions). Whiskers represent 1.5 times the interquartile range above the 75th percentile and below the 25th percentile.

As described above, in order to assess longer-lasting (i.e., “offline”) effects of LIFU, we also acquired ASL data prior to the first sonication run, in-between the two runs, and after the second run and performed an ROI analysis analogous to the one performed on the BOLD data. To assess this effect at the local level, we assessed if perfusion differed between these time points within the same 6 ROIs selected for the BOLD ROI analysis. Again, a four-way repeated measured ANOVA, as well as its Bayesian equivalent, including hemisphere (Left, Right), parameter set (Mode 1, Mode 2), time point (Pre LIFU 1, Post LIFU 1, Post LIFU 2) and ROI (Left Putamen, Left GP, Left Thalamus) as factors. As depicted in Fig. 3, we only observed a main effect of time point (*F*_*Greenhouse-Geisser*_ (1.332, 19.973) = 7.405, *p* = 0.008; BF_Inclusion_ = 127.923). Post-hoc analysis revealed that this effect was driven by a decrease in perfusion, within the ROIs, following LIFU 1 (*t*(15) = 3.651, *p*_*holm*_ = 0.003; BF_10_ = 9.23 × 10^4^), with no additional decrease observed following LIFU 2 (*t*(15) = 0.399, *p* = 0.844; BF_10_ = 0.10). No main effect of parameter set was found (*F*_*Greenhouse-Geisser*_(1,15) = 0.014, *p* = 0.908; BF_Inclusion_ = 0.015). An interaction between time-point and ROI was also observed (*F*_*Greenhouse-Geisser*_ (2.268, 34.013) = 4.503, *p* = 0.019) albeit with weak support (BF_Inclusion_ = 0.019), consistent with the fact that follow-up 2-way repeated measures ANOVAs—one per ROI—generally revealed the same pattern of decreased relative perfusion over time seen in the 3-way analysis (see supplementary materials for detailed report). As the main effect of time best characterizes the results across ROI’s extracted from ASL data, Fig. 3 (right) depicts data aggregated across all six ROIs.

## Whole brain analysis

### BOLD—whole brain block design

**Figure 4:**
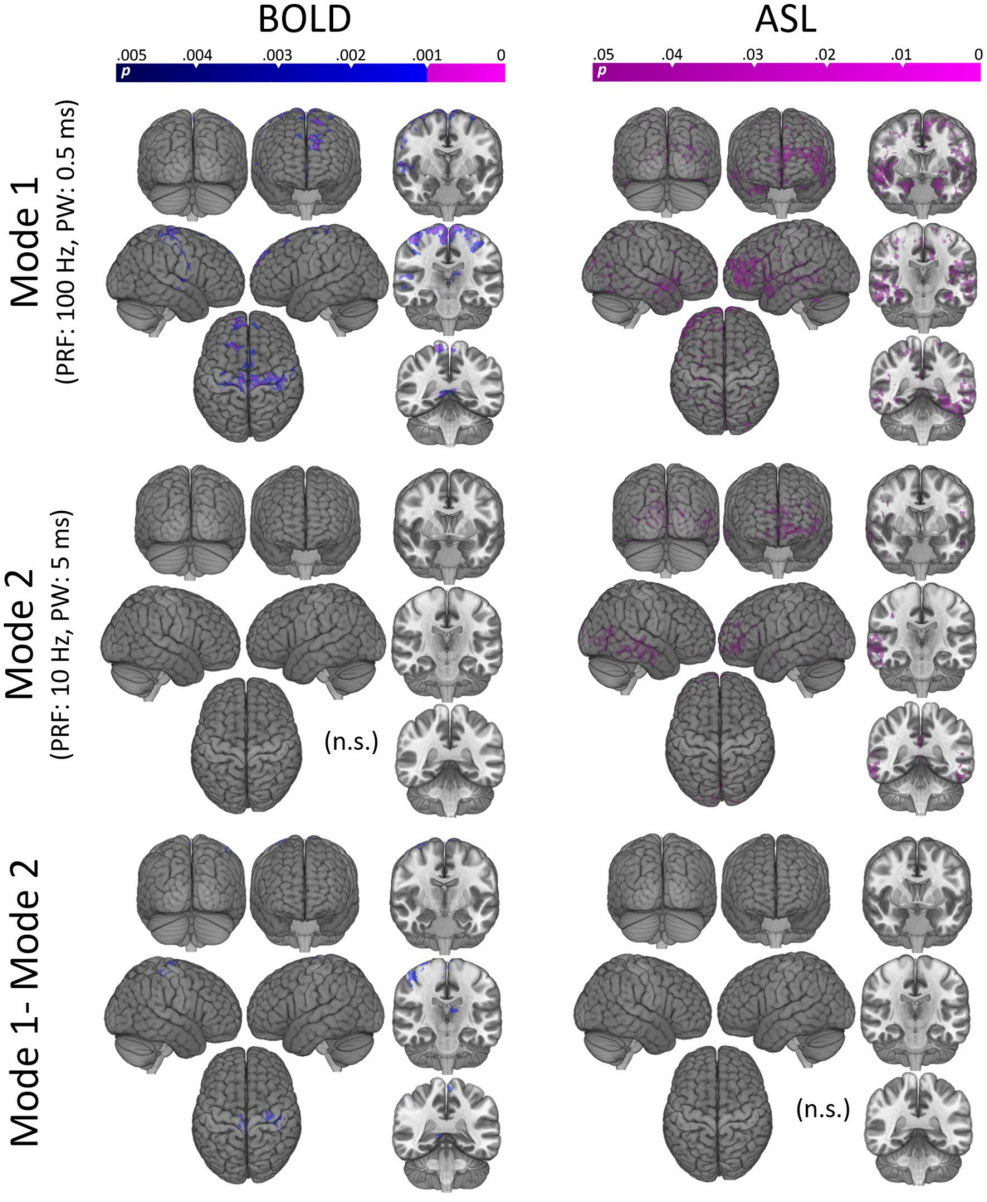
Whole brain results. Left: Regions of significant decrease in BOLD signal during sonication Mode 1 (top) and Mode 2 (middle) compared to inter-sonication periods (i.e., baseline). Subtraction of these results are also shown (bottom). Statistical maps were obtained using a mixed effects model (FLAME 1 + 2) as implemented in FSL^69^, and are shown at two levels of cluster correction for multiplicity (CDT set at *p* < 0.005, in blue, and at *p* < 0.001 in violet). Significant reduction in BOLD is found in several cortical regions, at both thresholds, for Mode 1. No regions of increased BOLD signal were observed for Mode 1. No regional increase or decrease was observed for Mode 2. Right: Regions of significant decrease in relative perfusion after sonication in Mode 1 (top) and Mode 2 (middle). Subtraction of these results are also shown (bottom). Statistical maps were obtained with a non-parametric approach (FSL randomise), as implemented in FSL, and are here shown at a level of *p* < 0.05 corrected for multiplicity with threshold free cluster enhancement (TFCE). No significant increase in ASL was observed for either sonication mode.

Full-brain analysis of the BOLD response during LIFU sonication (when compared to off periods) revealed several foci of reduced BOLD response during Mode 1 (PRF = 100 Hz, PW = 0.5 ms; see Fig. 4). Significant clusters included right and left pre- and post-central gyri, frontal polar cortex, posterior cingulate cortex, and Heschl’s Gyrus (see also Table S1). Given the known conservative bias of FSL-FLAME 1 + 2^30,31^ in single-sample t-tests at the employed cluster defining threshold (CDT; equivalent to *p* = 0.001; see Fig. 1A,B in ref 31^30^), results are shown at two CDT values (Fig. 4; *p* = 0.001, in violet, and *p* = 0.005, in blue). At this lower threshold, an additional cluster is visible in the medial frontal cortex and significant clusters expand to subsume portions of the dorsal thalamus. No area of increased BOLD response was observed at either CDT during LIFU Mode 1 sonication. Furthermore, no significant foci of increased or decreased BOLD signal were observed during LIFU Mode 2 (PRF = 10 Hz, PW = 5 ms) at either CDT, consistent with our ROI results.

Direct comparison of the BOLD response under the two stimulation modes revealed no significant differences at a CDT of *p* < 0.001. When displayed at the lower CDT (Fig. 4 bottom left), however, some foci of significant difference were observed in the very foci reported for Mode 1 (pre- and post-central gyri and posterior cingulate cortex/ left dorsal thalamus).

### ASL—whole brain

Given the ROI result, full-brain relative perfusion images were analyzed by contrasting the average brain perfusion after LIFU (i.e., the average of time-point 2 and time-point 3) to the relative perfusion prior to LIFU (as shown in Figure S4, the results are qualitatively unchanged when the data were analyzed with a linear model [i.e., time-point 3 versus time-point 1]). As shown in Fig. 4, a nonparametric permutation t-test, corrected for multiple comparisons with threshold-free cluster enhancement, TFCE^32^, revealed a broad decrease in perfusion throughout the cerebrum for both LIFU modes. In accordance with our ROI results, no significant difference was found when directly comparing sonication modes, and no increase in perfusion was found for either mode.

## Discussion

This study is the first demonstration of a group-wide^33^ MRI response from subcortical focused ultrasound in humans. Taken together, our results suggest that, when targeting the GP and adjacent structures: (1) LIFU, across two modalities (i.e., BOLD, ASL), in Mode 1 appears neuroactive both during (i.e., online) and after (i.e., offline) sonication. (2) The valence of online, offline, local, and distal effects all appear inhibitory (relative to baseline, for BOLD, and to pre-LIFU for ASL). (3) Different LIFU parameters (pulse repetition frequency and pulse width) indeed result in different online responses, as measured over seconds (30–60 s) with BOLD, but not in different offline effects, as measured over 10 s of minutes with ASL. Whether this reflects an additional temporal dimension to the parameter space of LIFU, remains to be understood.

Firstly, we asked if online LIFU could modulate BOLD signal in the target of interest as well as throughout the brain. We found an apparent inhibition of our intended target nuclei, as well as the adjacent left thalamus, during 30-s trains of LIFU in Mode 1 but not in Mode 2 (see Fig. 3). At the whole-brain level we also found an online inhibitory effect of LIFU in Mode 1, but not Mode 2, within the primary somatomotor cortex, left dorsal thalamus, as well as frontal and posterior cingulate association cortex concurrent with pallidal sonication (see Fig. 4, Table S1).

While GP structural connectivity remains elusive, the results presented here demonstrate a plausible pattern of effects. Inhibition of the left pallidum significantly affects all other components of the cortico-basal ganglia-thalamo-cortical circuit underlying motor refinement^13^ and cognitive functioning^14^, either by way of thalamus as conduit^14^ or through direct pallido-cortical projections^17–19,34^. Our BOLD results—primarily found in frontoparietal cortex and thalamic tissues—fit within this framework. Recording activity from both the target of interest and globally is key to understanding the valence of LIFU’s direct influence on neural tissue and the network effects this may bring about. Despite this, direct measurement from targeted subcortical structures is rare in LIFU studies (a notable exception being a recent study in macaques^9^) while it is completely absent in humans until this point, rather being inferred from downstream impacts on cortical activity^5,8,10^. Dual local and distal effects found here bolster the notion that fMRI can be used to effectively understand LIFU and its parameter space in healthy human subjects. On the contrary, if only distal effects were found, which is often the case in TMS-fMRI^35^, it would have severely impaired valence-specific conclusions about LIFU’s direct influence. These results, which are based on a the contrast of 30 s blocks of LIFU compared to 30 s inter-sonication baseline blocks, suggest that the effects of LIFU can vary over a time-course of seconds, despite our own findings in relative perfusion as well as prior results demonstrating sustained influence from LIFU lasting minutes^7^ or hours^9,36,37^. This finding supports the feasibility of classical block designs in future studies and invites investigation of online behavioral impacts from LIFU. Furthermore, our findings of pallidal as well as cortical (most relevantly in the primary somatomotor cortex) effects of pallidal LIFU also mirror the results of studies using contemporaneous neuroimaging and pallidal DBS^38,39^, potentially suggesting that this non-invasive technique might be employed in the future to evaluate patient suitability for invasive stimulatory procedures or as a treatment intervention itself.

In addition, we used ASL to characterize the relative longer-term effects of our sonication (i.e., minutes, rather than seconds following cessation). Unlike BOLD, which is derived from more complex interactions between blood flow and acute neural activity, ASL quantifies cerebral blood perfusion alone. This reduces the inter-subject and inter-trial variability of ASL relative to BOLD^40^, making ASL preferable for detecting disparate neural activity between distant time points. Here, we again found an apparent inhibition (decreased perfusion) of our target nuclei (and the adjacent putamen, thalamus) in the minutes following sonication, but it is important to note this inhibition was also observed in the right lateralized homologues of these structures, suggesting a more diffuse effect. Again, inhibition extended to the cortex, where decreased perfusion was found throughout the cerebrum (see Fig. 4). However, here, we find the same pattern of results following sonication in both Mode 1 and Mode 2, in contrast with our BOLD results.

These results corroborate other findings of an offline influence from LIFU^8,9,12,36,37^. However, the estimated focal intensities used here are approximately an order of magnitude lower than recent studies in pigs^8^ and macaques^9,36,37^. The time course of LIFU’s influence on neural activity remains unclear and appears to vary dramatically between experimental paradigms, ranging from seconds^41^ to over an hour^9,36,37^ with a trend towards long-term, offline effects occurring following longer sonication periods^12^. An offline impact may be expected here given that the 10 min of total sonication utilized far exceeded the 40 s necessary to elicit effects in the tens of minutes to hour range^9,36,37^, despite the lower intensities used in the present study. On the one hand, a significant perfusion effect of LIFU in Mode 2 (PRF = 10 Hz), despite null effects on BOLD data, suggests the heightened sensitivity of ASL compared to BOLD^40^ in this context. On the other hand, it may also suggest that LIFU parameters impact the time-course of LIFU’s neuromodulation, given the divergence in online (BOLD) and offline (ASL) results. Considering the recent offline behavioral effects reported in non-human primates^37^, our results further support the investigation of offline behavioral effects at intensities deemed safe for ultrasound imaging of the human cranium^42^, and its potential use in clinical applications seeking protracted impact on neural activity^20,43^. Why we observe a more diffuse effect in our ASL data, when compared to our BOLD data, can only be speculated on. It is conceivable, however, that such a broad effect may be mediated by a general reduction in arousal in the minutes following LIFU—a quality that the lentiform nuclei, and the circuits in which they participate, appear intimately involved in^14–16^.

Others have reported differential neuromodulatory effects from LIFU applied at different PRFs when intensity is held constant^41^; however, these differences are slight in comparison to alterations of other parameters, such as intensity or duty cycle^41^. LIFU parameters such as PRF are likely to interact with duty cycle, intensity, etc. in a high-dimensional parameter space that is far from fully characterized, as evidenced by divergent findings between studies using similar parameters (e.g., apparent disruption^8^ as well as excitation^7^ at PRF = 10 Hz with duty cycles near 50%). A possible explanation for these contrasting results is that LIFU modulation may be tissue-type selective. While the mechanisms underlying neuromodulation through LIFU remain debated, several theories highlight the possible interaction of mechanical pressure on neural tissue and lipid bilayer and/or membrane protein dynamics which brings about depolarization by altering membrane permeability or capacitance^44,45^. Tissue-specific effects are thus likely to be driven by differential expression of membrane proteins or the variable membrane dynamics between cell types. For instance, it has been proposed that inhibition through LIFU is achieved through preferential excitation of GABAergic interneurons rather than hyperpolarization of single cells^44,46^ and that this is the mechanism underlying a general trend of inhibition during lower duty cycles. While more work is needed to assess how LIFU interacts with the microstructure of the basal ganglia, these results support the notion of inhibition using low duty cycles, an important step towards clinical applications of LIFU.

The dimensions of the ultrasound beam produced with our single-element transducer ensure energy deposition into adjacent structures when targeting the GP due to its small size. This, indeed, is a limitation (as discussed below) in the use of LIFU for basic science of the GP alone. However, this may be of benefit to scientific and clinical applications aimed at broader basal-ganglia-thalamo-cortical^13,14^ circuits, whose subcortical components are ideally positioned for LIFU modulation through the temporal bone. Apparent inhibition of these circuits’ components here, if proved consistent, may make way for clinical use, with an emphasis on the modulation of basal-ganglia-thalamo-cortical^13,14^ communication that is presumed to underlie the efficacy of pallidal DBS in treating some individuals with obsessive–compulsive disorder^21^, Tourette syndrome^22^, treatment-resistant depression^23^ and movement disorders such as Huntington’s disease and Parkinson’s disease^24,25^. These results are also highly relevant to the continued study of LIFU’s influence in Disorders of Consciousness^20^ as DOC symptomatology has also been linked to basal-ganglia-thalamo-cortical commmuncation^14^ and GP atrophy specifically^47^. The properties of focused ultrasound suggest it may represent a sensible evolutionary step in obtaining subcortical neuromodulation in these applications, avoiding, for instance, the risks inherent to DBS—including hemorrhage and infection^48^—and the non-selectivity of pharmaceuticals thought to impact this system (e.g. zolpidem and amantadine^49^). These findings represent a pioneering step towards such applications as well as an early foothold for the use of MR-guided LIFU in human neuroscience more broadly.

Finally, interpretation of our data should be mindful of a number of limitations. Firstly, it is possible that changes in physiological arousal^50^ and its consequences (i.e., blood CO_2_ content^51^, and blood pressure^52^), might affect BOLD and ASL data analysis. Similarly, higher intensity LIFU can induce vasoconstriction^53^, which could alter BOLD and ASL data. However, it remains to be seen if this is relevant to the low intensities used here. In addition, LIFU has been shown to suffer from potential confounds including MRI dropout^33^, due to the introduction of the transducer in the MRI environment, as well as auditory stimulation^54^, due to either the transducer producing sounds during pulsation and/or bone conduction effects. Although we cannot fully discount either possibility, a number of observations disfavor both. With respect to MRI dropout, the transducer was present during all functional acquisitions, so while it might lead to greater noise, it cannot explain either the time-varying BOLD result or the differential effects observed across modes of sonication. Furthermore, any decrease in signal-to-noise ratio due to such dropout acts in the direction opposite to our results. With respect to the possibility of LIFU effects being mediated by sound perception, this seems inconsistent with the observed decrease in both BOLD signal and perfusion (ASL) following sonication. Furthermore, although not originally predicted, sonication in Mode 2 acts as a negative control for the findings observed with Mode 1, also suggesting that our results are specific to the parameters employed and not simply a consequence of artifact (i.e., MRI, and/or auditory), expectation effects, or inflated type I error^30^. Second, it should be appreciated that because of the comparative nature of BOLD analysis^55^, we cannot distinguish the degree to which the result observed during Mode 1 sonication is due to BOLD depression during stimulation and/or “rebound” activation during rest intervals in between sonication blocks.

Third, while we make use of subject-specific MR data and iterative assessments to guide LIFU positioning and targeting, the numerical simulations reported above (cf., Figs. 1B–D, 2) confirm the known limitations in the focal precision of LIFU with single-element transducers. On one hand, radially, LIFU demonstrates a precision on the order of 0.5 cm at its focus of maximal stimulation, which compares favorably with other non-invasive techniques such as TMS and tES. On the other hand, longitudinally, we observe a characteristically elongated focus, in the order of 1.5 cm, in stark comparison to the more precise focus achievable with multi-unit arrays^56^. Ultrasound energy deposition in this experiment was thus likely to be localized to the pallidal target as well as the adjacent nuclei of the putamen and thalamus, as well as the large white-matter tracks that surround them. This limitation complicates the placement of these results into canonical network models; for, thalamic and striatal function might be influenced both indirectly, through the modulation of pallidal output, as well as directly, through some degree of LIFU modulation. Similarly, direct modulation of axonal projections^44,45^ of the large white matter tracts intersected by the ellipsoid of maximal acoustic energy (e.g. the internal capsule) is also possible, but hard to characterize with the collected data^57^.

Finally, skull-induced variability and attenuation of acoustic energy remains another important limitation in human LIFU research. Corroborating other findings^10,27,58^, numerical modeling performed here through a high-resolution CT image (Visible Human^28^) suggests a general preservation of the ultrasound waveform observed in free water^10,27^. However, the degree of preservation in beam shape is likely to vary between individual sessions due to variable positioning, individual skull morphology and individual skull density^27^, the latter of which cannot be readily assessed using MRI at this time. As a consequence, we cannot readily evaluate the degree to which our sonication encompassed only the lateral segment of the GP (i.e., its pars externa) or both the lateral and medial segments (i.e., pars externa and pars interna). Yet, these two subregions of the GP are known to have different innervations^19^ and could lead to different patterns of cortical modulation, further complicating the full circuit-level interpretation of our findings and highlighting the possible need for subject specific numerical modeling in future investigations.

## Methods

### Participants

Participants included 16 healthy individuals (15 male; age = 18–44 years (M = 25.25; SD = 7.78)). Participants were screened for eligibility, reporting no history of neurological/psychiatric disorder or medical condition that may preclude safe entry into an MR environment. Participants were instructed to have the hair around their left temple below 0.5 inches during LIFU sessions due to the potential of hair to trap air bubbles and attenuate ultrasound. This requirement resulted in a high proportion of males. Participants received $150 compensation for taking part in the experiment. Written informed consent was obtained from all subjects according to the procedures approved by the UCLA Institutional Review Board. All procedures were performed in accordance with relevant guidelines and regulations. Informed consent was obtained for publication of the identifying information/images of the participants.

### Experimental design and MR image parameters

All participants underwent three discrete sessions in the 3 T Siemens Prisma Magnetic Resonance Imaging (MRI) scanner at the Staglin IMHRO Center for Cognitive Neuroscience at UCLA. For each session, a 20-channel siemens Prisma head coil was used in order to allow the space necessary for our transducer. During **session (1)** baseline structural and functional BOLD data was collected. This included a T1-weighted structural sequence (MPRAGE, TR = 2000 ms, TE = 2.52 ms, voxel size 1 mm^3^), T2-weighted structural sequence (TR = 1500 ms, TE = 104 ms, voxel size 1 mm^3^), a baseline arterial spin labeling image (TR = 4.6 ms, TE = 16.18 ms, 8 sequential slices, voxel size 1 mm^3^, inversion time = 1990 ms, tag-controlled pulsed ASL (PASL), bolus duration = 700 ms), a baseline functional image (T2*-weighted Gradient Recall Echo sequence, TR = 700 ms, TE = 33 ms, FOV dimensions: [78, 78, 54 voxels], voxel size 2.5 mm^3^, interleaved multiband acquisition, 1000 volumes total), and a white-matter nulled T1-weighted image used to better capture the fine structure of subcortical nuclei. During **sessions (2) and (3)** LIFU was applied**.** Firstly, a high-resolution T1-weighted MPRAGE sequence (TR = 2000 ms, TE = 2.52 ms, voxel size 1 mm^3^) was acquired for data analysis (i.e., registration of functional data from native space to the MNI template). Then, an additional lower-resolution T1-weighted image (TR = 1900 ms, TE = 2.2 ms, voxel size 2 mm^3^) was acquired following transducer placement to confirm final transducer positioning (see “Ultrasound Positioning” section). Once the transducer was positioned, participants underwent 5 functional acquisitions (with the same parameters as in the first session): two T2*-weighted BOLD sequences, acquired contemporaneously to LIFU sonication, and three ASL sequences, collected prior to the first LIFU run, after the first LIFU run (i.e., in between LIFU runs 1 and 2), and after the second LIFU run. In other words, participants underwent 5 functional scans in the following order: ASL (i.e., “Pre-LIFU 1”), BOLD-LIFU (i.e., “LIFU run 1”), ASL (i.e., “Post-LIFU 1”), BOLD-LIFU (i.e., “LIFU run 2”), and ASL (i.e., “Post-LIFU 2”). In each LIFU run, 5 min of sonication were administered in 10 off–on cycles consisting of 30-s blocks of LIFU preceded by 30-s blocks of rest (resulting in 10 “LIFUP-off” and 10 “LIFUP-on” blocks of 30 s each; see Fig. 1). Finally, in Session 3, participants underwent again the same data acquisition (and order). Sessions 2 and 3 thus only differed in the sonication parameter set used. Specifically, half the participants underwent two Mode 1 sonications in Session 2 and two Mode 2 sonications in Session 3, whereas the remaining participants underwent two Mode 2 sonications in Session 2 and two Mode 1 sonications in Session 3. Order was counterbalanced across participants.

### Ultrasound positioning

During sessions 2 and 3, the ultrasound transducer was positioned so that its center lay on the left temple (approximately 1/3 of the distance from the corner of the left eye to the left tragus for each participant and superior 2 cm). Ultrasound gel (aquasonic) was firstly applied to this region in an area subsuming the diameter of the transducer and rubbed into any hair present so that no hair permeated the gel layer in order to minimize air bubbles and ensure a smooth surface for coupling. A thin layer of gel was applied to the surface of the transducer and bubbles were similarly smoothed from this layer. The transducer was then coupled to the head with gel filling any concavity between the transducer membrane and the scalp. Two straps—one horizonal and one vertical—secured the device to the participant (see Figure S6). Next, we acquired a rapid (95 s) T1-weighted structural sequence (TR = 1900 ms, TE = 2.2 ms, voxel size 2 mm^3^). Using a circular MR fiducial and the visible center of the transducer, reference lines were drawn using the scanner console in the transverse and coronal planes to locate the target of the LIFU beam visually in three dimensions. Adjustments to the positioning were made iteratively until the trajectory of our ultrasound beam passed through the temporal bone, through the anterior dorsal aspect of the left globus pallidus transversely as well as coronally and terminating into the left thalamus. This portion of the GP was chosen for its apparent direct structural connectivity with frontal cortex^19^ and our interest in the role of the GP in cognitive functioning. The dimensions of the -3 decibel focus of our transducer when measured in water (see Fig. 1) and simulated through bone (see Fig. 2), as well as the orientation of the ultrasound beam, suggests that this targeting ensures the beam consistently subsumes aspects of the left GP, with the adjacent left putamen and left thalamus also likely impacted directly by LIFU. Following each sonication, a rapid (95 s) T1-weighted structural sequence (TR = 1900 ms, TE = 2.2 ms, voxel size 2 mm^3^) was always taken to ensure the transducer remained aimed towards our target. It was our intent to repeat a session if such an event occurred; however, it was never necessary to do so as large movements of the transducer did not occur during sonication.

### Ultrasound waveform

A BXPulsar 1001, Brainsonix Inc. ultrasound device was used in two modes. While both modes employ a 650 kHz carrier wave, the distinction between them is a high PRF (100 Hz) with low Pulse Width (0.5 ms) for Mode 1 and a low PRF (10 Hz) with high Pulse Width (5 ms) for Mode 2. Pulsation was administered in 2, 10-min sessions on each of 2 different days and thus in 4, 10-min sessions total. Within each 10-min session, pulsation alternated between 30 s of LIFU and 30 s of no-LIFU for a total of 10, 30 s trains of pulsation per sonication (5-min of LIFU total per 10-min session)—a standard block design amenable to BOLD MRI. For both modes, carrier wave frequency = 650 kHz, Duty Cycle = 5%, I_spta.3_ = 720 mW/cm^2^; I_sppa.3_ = 14.40 W/cm^2^ (see Fig. 1A). This intensity falls under the safety guidelines for ultrasound imaging of the cranium provided by the FDA^42^.

### Ultrasound simulation (numerical modeling)

We simulated the propagation of the ultrasound waveform through a human skull using acoustic wave equations implemented in MATLAB using k-Wave (v1.1)^26^, a k-space pseudospectral solver toolbox. We applied these equations to computational head models derived from imaging data from the Visible Human Project^28^, provided courtesy of the U.S. National Library of Medicine. Specifically, we used the head CT (dimensions: 512 × 512 × 512 voxels) and its paired T1-weighted MRI (dimensions: 196 × 231 × 67 voxels) data from the Visible Human Male (VHM) dataset^28^. The VHM MRI data were aligned to the VHM CT using FSL’s FLIRT program (6DOF model) and resampled to the voxel grid of the CT data. We manually defined a target beam trajectory through the temporal bone and into the left pallidum on a reference MRI brain atlas (MNI152, 1 mm). This enabled improved identification of relevant anatomy for landmark selection as compared to the Visible Human dataset’s male MRI, which is of relatively low resolution. The trajectory was defined by selecting two anatomical points in the MNI152 atlas: (1) a target point in the anterodorsal pallidum, at which the ultrasound was aimed from the left side of the head; and (2) a point denoting the center of the transducer’s face outside the head at the point of contact with the skin. Care was taken to ensure the transducer’s membrane was flush with the skull, as was the case in our experimental setting. We registered the MNI152 atlas to the transformed VHM MRI data using FLIRT (12 DOF model) and used this transform to map the target and transducer points to the space of the VHM CT data.

Head models were derived from the CT data as follows. We first resampled the CT data into a 512 × 512x512 volume with isotropic voxels (0.489 mm × 0.489 mm × 0.489 mm) using trilinear interpolation. This defined the simulation grid, whose dimensions enabled us to take advantage of the speed offered by the Fast Fourier Transform used in k-Wave's Fourier collocation method when calculating spatial gradients. We generated three 3D arrays based on the CT scan that modeled the 1) density, 2) speed of sound, and 3) nonlinearities of the varying materials (e.g., scalp, skull, brain) at each position in the grid. These values were derived using mappings of CT intensity, based on Hounsfield unit values, to corresponding values for medium density, speed of sound, and absorption using the porosity method, as performed by Legon et al.^10^.

These rectilinear grids were then used to simulate LIFUP from a fixed simulated transducer on the subject’s head. We modeled a 72 mm diameter single-element transducer positioned in the grid based on the transformed transducer point. We defined a focal point tracing forward 55 mm (the length of our transducer’s focal depth from its membrane) along the line from the transducer point to the pallidal target point. Wave propagation from the transducer converging toward this focal point was simulated in the grid. We modeled the pressure wave emitted from the transducer as a three-dimensional sinusoidal wave using parameters obtained from water tank experiments (Acetera), specifically including a pressure at the face of the transducer of 1.0558 MPa and a carrier frequency of 650 kHz. We simulated the propagation of this pressure wave from the transducer by solving coupled 1st-order nonlinear differential equations on the rectilinear grids of values representing density and speed of sound. The combination of these equations yields the generalized form of the Westervelt equation^59^. We solved this system of equations using k-Wave^26^. The maximal pressure and particle velocity values encountered at every voxel over time in the rectilinear grid simulation domain were recorded for analysis.

This scheme was simulated for a length of time corresponding to one pulse of the mode 2 parameter set used here (see ultrasound waveform for a complete description of parameter sets). In the mode 2 (10 Hz Pulse Repetition Frequency) scheme, our pulse is on for 0.005 s and off for 0.095 s (0.1 s Pulse Period for a duty cycle of 5%). We thus simulated a duration of 0.005 s, or 3250 cycles at 650 kHz (Carrier Frequency). We performed the simulation twice, first using the CT-derived head model and then using a free-water model, applying the same trajectory in both cases. For each simulation, the peak intensity inside the brain (the majority of energy deposition is in the skull when it is present) as well as its voxel coordinate were captured. We calculated the distance between the simulated point of highest intensity in free water and through bone in order to capture the degree of refraction expected to occur due to propagation through skull. Similarly, the peak intensity in both free-water and CT simulations were compared in order to quantify the degree of peak pressure attenuation expected due to skull. The cumulative energy deposition over time was calculated for the voxel of peak intensity for the through-skull simulation. Energy deposition over time was highly linear, suggesting that the shorter pulse width used here, 0.5 ms, in terms of energy deposition, behaves the same as the longer pulse (see Figure S5) and that the results presented generalize to both pulse widths.

## BOLD data analysis

Data analysis was conducted using FSL (FMRIB Software Library v6.0.1)^60^ with in-house Bash shell scripts. In addition, second level data analysis was performed using JASP. JASP Team (2019). JASP (Version 0.11.1).

### Preprocessing

Prior to analysis, preprocessing was performed. This included brain extraction of T1 images (optibet^61^). Functional data was preprocessed using motion correction (MCFLIRT)^60,62^, slice timing correction (Fourier-space time-series phase-shifting), spatial smoothing (using a Gaussian kernel of 5 mm full-width half-max), and high-pass temporal filtering (Gaussian-weighted) at 0.01 Hz. In this study, the mean relative displacement was 0.25 mm while the mean absolute displacement was 0.28 mm. Of the 64 collected BOLD datasets, none exhibited an absolute motion exceeding 2.5 mm (voxel size = 2.5 mm isovoxel) or relative motion exceeding 1.25 mm.

Registration from functional to structural space for each subject was achieved using FSL’s Boundary-Based Registration (BBR)^63^, with the exception of two subjects for whom linear (FSL FLIRT^60,62^; 12 degrees of freedom) resulted in better functional-to-structural alignment. Finally, T1-weighted data were co-registered to the MNI template using FSL FNIRT^60^ (i.e., 12 dof linear coregistration followed by non-linear warping [Warp resolution of 10 mm]). Satisfactory registration was confirmed visually for each run.

### BOLD—whole brain block design

LIFU-BOLD data sequences were first analyzed employing a univariate general linear model (GLM) approach^64^ including a pre-whitening correction for autocorrelation (FILM). For each LIFU-BOLD sequence for each participant, a univariate analysis was conducted using a single “task” regressor—onset time of 30 s blocks of LIFU administration; moreover, 24 extended motion regressors were employed, including motion in 6 directions as well as first derivatives, second derivatives, and their difference. Thus, here, “baseline” refers to inter-sonication periods where no LIFU is applied. For each BOLD sequence, we computed 2 contrasts: LIFU > no LIFU and LIFU < no LIFU. Data from LIFU Mode 1 and data from LIFU Mode 2 were aggregated respectively and assessed statistically using a mixed effects FLAME 1 + 2 model. At level 2, these results were aggregated between runs of the same LIFU parameter set while the following contrasts were calculated on a per-subject basis: Mean LIFU Mode 1, Mean LIFU Mode 2, LIFU Mode 1—LIFU Mode 2, LIFU Mode 2—LIFU Mode 1, LIFU Mode 1 + LIFU Mode 2. At level 3, data were aggregated between subjects. Data were cluster corrected for multiple comparisons using a cluster-level threshold of z > 3.09 (corrected *p* < 0.05). A separate level 3 analysis was conducted with cluster correction at z > 2.57 (corrected *p* < 0.05).

### BOLD—ROI

Based on the trajectory of our ultrasound beam, three subcortical ROI’s (Left Putamen, Left Globus Pallidus and the Left Thalamus) were selected to determine if FUS modulated the BOLD signal in targeted regions and/or in adjacent regions. As a control, right lateralized structures were also selected (Right Putamen, Right Globus Pallidus and the Right Thalamus). For each subject, masks for each region were created by using FSL’s automatic segmentation for subcortical nuclei (FIRST)^60^ for subcortical extraction on high resolution T1 images. Each subcortical ROI was binarized and confirmed for correct extraction visually. For each statistical z-score map produced in the GLM contrast of no LIFU blocks (see Block Design above) with LIFU blocks (LIFU > no LIFU), voxel-wise z-scores were averaged inside the area of each ROI using FSL’s tool for extracting the mean selected voxels in a 3D image (fslmeants)^60^. Registration via FSL’s linear registration tool (FLIRT^60,62^, Normal search, 12 DOF) of each z-score map to structural masks was confirmed visually. This leaves us with one number denoting the z-scored difference in BOLD between LIFU-on and baseline (LIFU-off) within each ROI for each LIFU run for each subject. Percent signal change (See Figure S7) was quantified by each region by using FSL’s featquery command^60^. When computing percent signal change, ROI’s were extracted at the third level FEAT using the Harvard–Oxford subcortical atlas.

A 2 × 2 × 2 × 3 repeated measures ANOVA was performed with Hemisphere (Left, Right), Parameter Set (Mode 1, Mode 2), Run (Run 1, Run 2), and ROI (Left Putamen, Left GP, Left Thalamus) as factors. Follow-up 2 × 2 × 3 repeated measures ANOVAs were performed for each hemisphere with Parameter Set (Mode 1, Mode 2), Run (Run 1, Run 2), and ROI (Left Putamen, Left GP, Left Thalamus) as factors. Follow-up 2 × 2 repeated measures ANOVAs were performed for each Left-Lateralized ROI with Parameter Set (Mode 1, Mode 2), Run (Run 1, Run 2) as factors. In each of these follow-up two-way ANOVAs, marginal means were computed for each parameter set and statistically assessed against zero in order to assess if an influence from each parameter set existed irrespective of the other. Šidák correction was used to correct for multiple comparisons across marginal mean tests. Mauchly’s test of sphericity was found to not have been violated. The Shapiro Wilk test confirmed normality in the data. Several outliers existed in this data. A separate analysis was conducted with outliers excluded; this did not change results and so outliers were left in the data presented here.

In addition to frequentist testing, Bayesian repeated measures ANOVAs were performed alongside all frequentist ANOVAs and which used the same factors. Marginal Means were assessed as Bayesian t-tests. Bayesian one-sample one-sided t tests were conducted for each parameter for each ROI against zero in which the alternative hypothesis stated the data was below zero. For all Bayesian t-tests employed throughout this study, a Cauchy distribution with a width parameter of 0.707 was used while, for all Bayesian ANOVAs, a r scale prior width of 0.5 was used; see JASP documentation for a discussion of Markov chain Monte Carlo (MCMC) settings, which are determined in a black-box manner by the JASP program. Bayes factors (BF) reported reflect the ratio of evidence for each alternative hypothesis (H1) against the null hypothesis(H0). A BF_10_ indicates the Bayes factor in favor of H1 over H0. A BF_10_ > 3 is widely considered as positive and substantial evidence for the alternative hypothesis^65^. BF_Included_ denotes the evidence in support of the inclusion of effects (e.g., interaction terms) in the model over and above that of other terms. In order to estimate BF for marginal means, a separate analysis was performed in which runs were averaged. Statistical analysis was performed in JASP.

## Arterial spin labeling (ASL)

### ASL—whole brain

In order to quantify the degree of blood perfusion throughout the brain at three time points for each subject visit—pre LIFU 1, post LIFU 1, and post LIFU 2—each ASL sequence was analyzed using standard processing methods in FSL Bayesian Inference for Arterial Spin Labeling MRI^66^ (BASIL), using the command-line tool oxford_asl. Analysis was set to conform to the assumptions concerning the kinetic model and T1 values outlined in the BASIL white paper^66^. The standard T1 value of atrial blood (T1b) was used (1.65). The inversion efficiency of pASL was set at 0.98. Estimation of bolus duration was disabled and supplied at 700 ms. Spatial regularization^67^ as well as motion correction^60,62^ was used while artifact correction for ASL signal within the microvasculature was disabled. The resultant perfusion images were registered to standard space; the quality of these registrations was confirmed visually. At level 2, two linear and two “L” models were applied to the three estimates of perfusion derived from level one, for each mode (10 Hz vs. 100 Hz) and for each subject. One linear model described a descending trend in blood perfusion through the session (1 0 − 1) while the other, an ascending trend (− 1 0 1). One “L” model described a descending trend in blood perfusion after LIFU 1 but decreasing no further (1 -0.5 -0.5) while the other, an ascending trend after LIFU 1 but then increasing no further (− 1 0.5 0.5). At level 3, data was aggregated within respective modes and across subjects as well as statistically assessed using FSL randomise’s^68^ nonparametric permutation t test for voxel-based thresholding. A single-sample two-sided t test was run with threshold free cluster enhancement (TFCE)^32^, correcting the resultant *p*-values for multiple comparisons across space. The “L” model is presented in the main results section.

### ASL—ROI

The ROI’s investigated in BOLD were also analyzed using ASL data. These included the Left Putamen, Left Globus Pallidus, and Left Thalamus. The same masks used for the BOLD ROI analyses (see Block Design BOLD ROI) were used here. Each perfusion image was registered to subject structural space using oxford_asl. Intensity of perfusion images were averaged inside the area of each ROI using FSL’s tool for extracting the mean selected voxels in a 3D image (fslmeants)^60^ for each perfusion image for each subject (16 images; 3 time points per 2 parameter sets).

The resultant intensity values were run firstly in a 2 × 2 × 3 × 3 repeated measures ANOVA with Hemisphere (Left, Right), Parameter Set (Mode 1, Mode 2), Time Point (Pre LIFU 1, Post LIFU 1, Post LIFU 2) and ROI (Left Putamen, Left GP, Left Thalamus) as factors. A “Repeated” contrast was run for Time Point, which directly compares Pre LIFU 1 with Post LIFU 1 and Post LIFU 1 with Post LIFU 2. Follow-up 2 × 3, two-way repeated measures ANOVA’s were run for each ROI with Parameter Set (Mode 1, Mode 2) and Time Point (Pre LIFU 1, Post LIFU 1, Post LIFU 2) as factors. A “Repeated” contrast was run for Time Point, which directly compares Pre LIFU 1 with Post LIFU 1 and Post LIFU 1 with Post LIFU 2 for each of these follow-up tests. Mauchly’s test of sphericity was found to be violated in several of these ANOVAs, thus the Greenhouse–Geisser correction for sphericity was applied to these. The Shapiro Wilk test confirmed normality in the data. No outliers were found in these data.

As per the ROI analysis performed on BOLD data, Bayesian repeated measures ANOVAs were run with the same factors. In order to estimate BF for “Repeated” models, a follow-up comparison between Time Points was conducted. Statistical analysis was performed in JASP.

## Supplementary Information

Supplementary Information

## Data Availability

Anonymized data are available from the first (joshcain@ucla.edu) or last (monti@ucla.edu) author and can be obtained through an MTA between the requesting PI/institution and the UCLA Technology Development Group (TDG).
