# Supplementary material for: Real time and delayed effects of subcortical low intensity focused ultrasound
Source: Sci Rep. 2021 Mar 17;11:6100. doi: 10.1038/s41598-021-85504-y (PMC7969624; doi:10.1038/s41598-021-85504-y)
Supplement: Supplementary file 1 — Supplementary Information [file 41598_2021_85504_MOESM1_ESM.pdf]

Real Time and Delayed Effects of Subcortical Low Intensity Focused Ultrasound

Joshua A. Cain[1]*, Shakthi Visagan[2], Micah A. Johnson[1], Julia Crone[1], Robin Blades[1,2], Norman M. Spivak[4,5], David W. Shattuck[2], and Martin M. Monti[1,3]

[1]Department of Psychology, University of California Los Angeles, Los Angeles, 90095, USA

[2]Department of Neurology, University of California Los Angeles, Los Angeles, 90095, USA

[3]Department of Neurosurgery, University of California Los Angeles, Los Angeles, 90095, USA

[4]Department of Psychiatry, University of California Los Angeles, Los Angeles, 90095, USA

[5]Brain Injury Research Center (BIRC), Department of Neurosurgery, University of California Los Angeles, CA, 90095

* Correspondence should be addressed to: Josh A. Cain (joshcain@ucla.edu). UCLA Department of Psychology, Pritzker Hall, Los Angeles CA 90095.

Table of Contents

- **Supplemental Tables………………………………………………………………………………..Page 3**
  - **Page 3…Table S1: BOLD Changes in Whole-Brain During 100Hz Sonication Compared to Baseline**
- **Supplemental Figure……………………………………………………………………………Page 4-11**
  - **Page 4…Figure S1: Aggregating LIFU Mode 1 and LIFU Mode 2 data**
  - **Page 5…Figure S2: Comparisons between LIFU1 and LIFU2 for LIFU in Mode 1 (PRF = 100Hz)**
  - **Page 6… Figure S3: Comparisons between LIFU1 and LIFU2 for LIFU in Mode 2 (PRF = 10Hz)**
  - **Page 7… Figure S4: Whole-Brain Perfusion Linear Model**
  - **Page 8… Figure S5: Cumulative Pressure Over Time; Figure S6: Ultrasound Stabilization**
  - **Page 9… Figure S7: BOLD ROI Percent Change in Signal**
  - **Page 10… Figure S8: Study-Wide Averaged HRF**
  - **Page 11… Figure S9: Pearson's Correlation between Left Globus Pallidus and Modulated Regions**
- **Supplemental Results…………………………………………………………………………..Page 12-13**
- **Supplemental Methods……………………………………………………………………..…..Page 14-15**
- **References…………………………………………………………..………………………….Page 16**

SUPPLEMENT

Region	Voxels	Z-Max	Z-Max (MNI)		
			x	y	z
LIFU Mode 1 – Baseline					
*Midline Precentral Ctx.	4127	5.01	4	-18	76
*Posterior Cingulate Ctx.	855	4.1	6	-40	0
*Heschel's Gyrus	484	3.71	60	-6	8
*Frontal Polar Ctx.	457	4.15	-2	62	32
Frontal Medial Ctx.	234	3.6	-4	46	-16
LIFU Mode 1 – LIFU Mode 2					
Midline Postcentral Gyrus	510	4.31	38	-34	64
Midline Precentral Gyrus	245	4.04	-4	-30	74
Posterior Cingulate Ctx.	218	3.87	8	-40	0

Table S1: BOLD Changes in Whole-Brain During 100Hz Sonication Compared to Baseline, 10Hz Sonication. Significant clusters as defined by a cluster significance level of $p < 0.05$ with a cluster defining threshold (CDT) of $p < 0.005$. Regions that also survived a more conservative CDT of $p < 0.001$ are marked with an asterisk *.

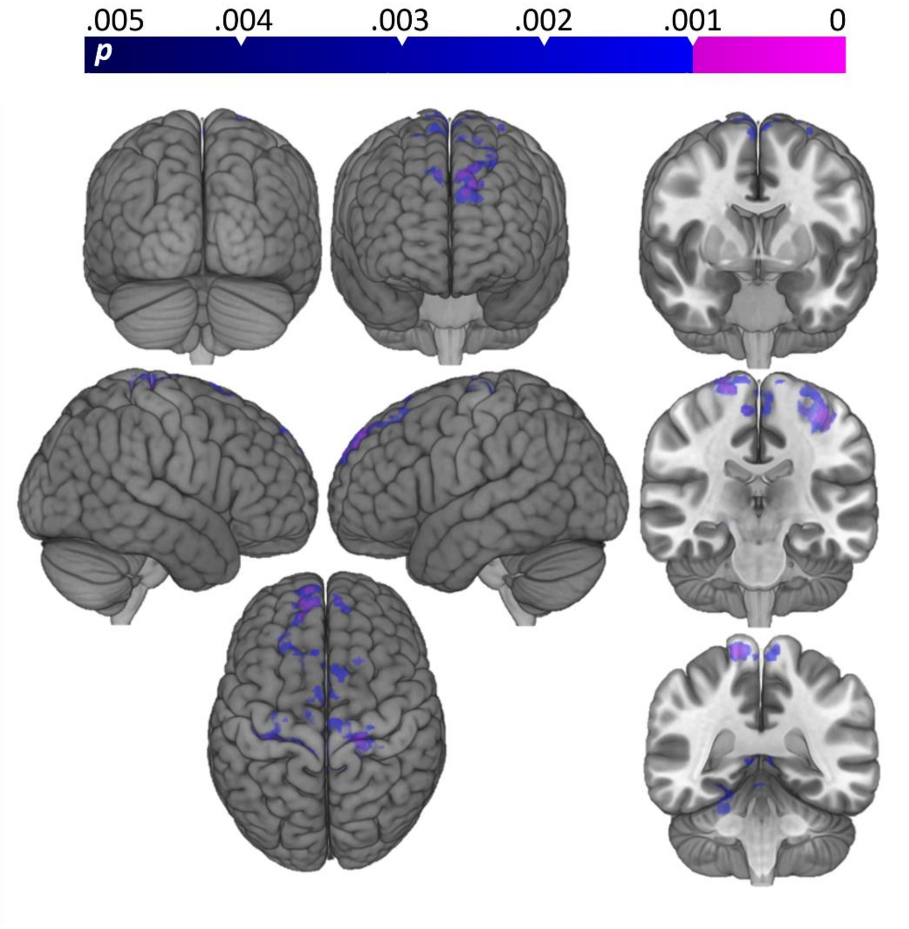

Figure S1: Aggregating LIFU Mode 1 and LIFU Mode 2 data. Shown here are the results of modeling our block design on data captured during both LIFU modes (Mixed Effects FLAME 1+2; cluster significance: $p < 0.05$; cluster defining threshold: $p < 0.005$ (blue), $p < 0.001$(violet)). Despite the boost in statistical power theoretically provided by doubling the data included, no new clusters are added to the results obtained when analyzing LIFU Mode 1 alone.

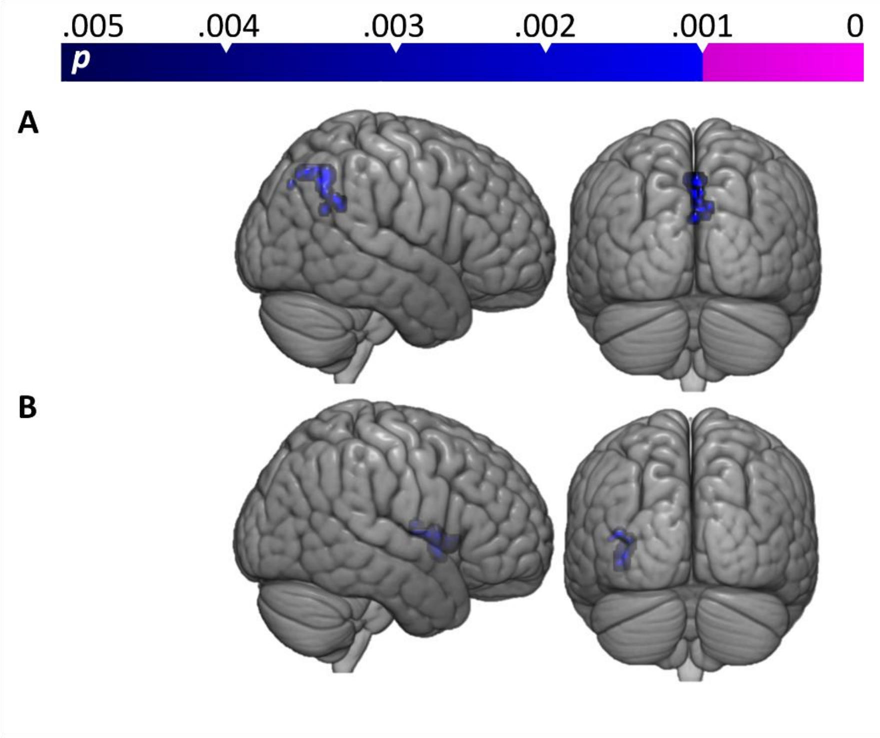

Figure S2: Comparisons between LIFU1 and LIFU2 for LIFU in Mode 1 (PRF = 100Hz). Shown here is a statistical map (Mixed Effects FLAME 1+2; cluster significance: $p < 0.05$; cluster defining threshold: $p < 0.005$ (blue), $p < 0.001$(violet)) of results obtained when subtracting successive LIFU runs (two data sets were collected per session) during Mode 1 (PRF = 100Hz) LIFU**. A)** LIFU1 -LIFU2 **B)** LIFU2-LIFU1**.** Significant voxels denote regions of relatively decreased BOLD (i.e., more suppression of BOLD signal from baseline). Minor differences exist, suggesting no major sensitization or habituation to LIFU's influence.

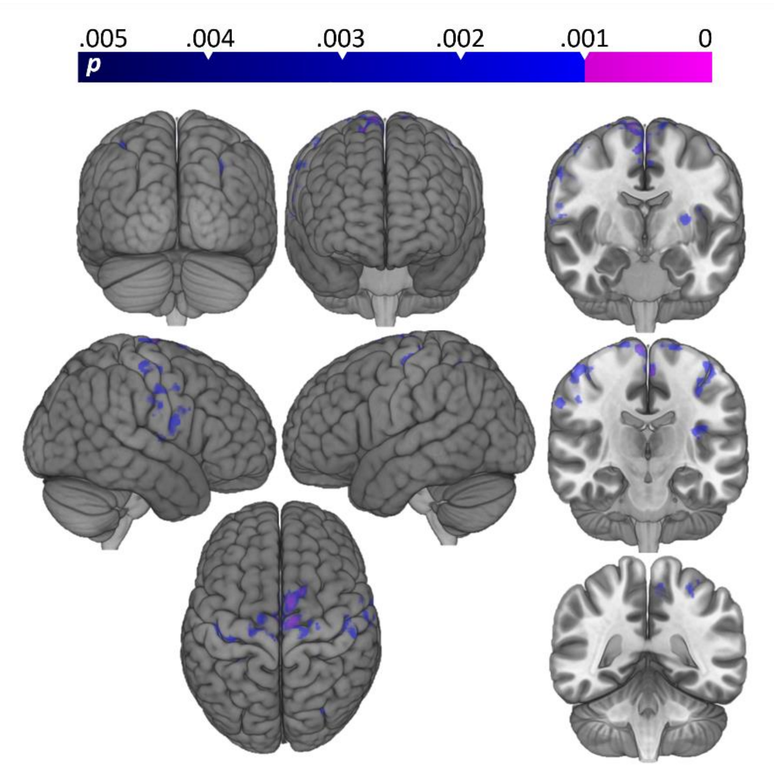

Figure S3: Comparisons between LIFU1 and LIFU2 for LIFU in Mode 2 (PRF = 10Hz). Shown here is a statistical map (Mixed Effects FLAME 1+2; cluster significance: $p < 0.05$; cluster defining threshold: $p < 0.005$ (blue), $p < 0.001$(violet)) of results obtained when subtracting successive LIFU runs (two data sets were collected per session) during Mode 2 (PRF = 10Hz) LIFU**.** Significant voxels denote regions of relatively decreased BOLD (i.e., more suppression of BOLD signal from baseline). We find a greater inhibition of BOLD signal in LIFU 2 compared to LIFU 1, in regions generally in line with inhibition found when comparing LIFU Mode 1 to baseline, suggesting that perhaps a sensitization occurs during LIFU in Mode 2 over time.

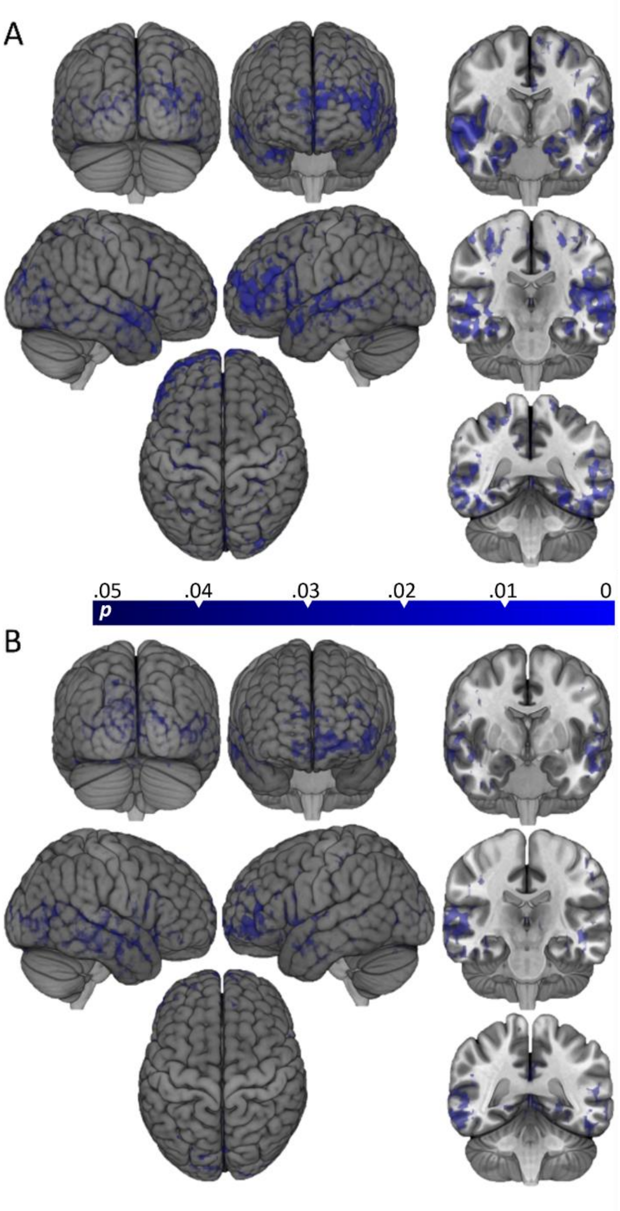

Figure S4: Whole-Brain Perfusion Linear Model. Statistical maps of Blood Perfusion resulting from ASL analysis for all subjects (n=16), including 3 total ASL captures per subject per parameter set—taken immediately before and after each pallidal LIFU in **A)** Mode 1 (100Hz PRF; 5ms PW) and **B)** Mode 2 (10Hz PRF; 0.5ms PW). Color voxels indicate those that fit a linear shaped model such that activity decreased following sonication 1 and decreased further following sonication 2. The colored bar indicates the p-value window with $p < 0.05$. No increase in blood perfusion at these parameters is indicated because none was found. No subtraction between parameters is shown because none was found.

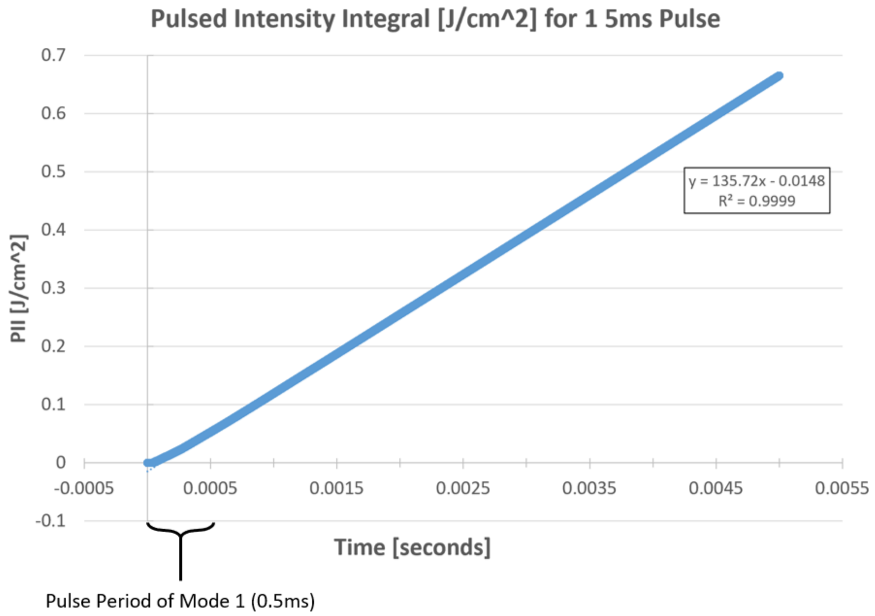

Figure S5: Cumulative Pressure Over Time. Results from monitoring the pressure at the voxel of maximum intensity (see Figure 2) when simulating a 5ms pulse over the full course of that pulse. The simulation scheme is the through-skull scheme described in Methods. As demonstrated by an R^2 of effectively 1, the pressure experienced by the brain appears highly linear over the time period of this pulse. This suggests a highly similar pressure distribution over-time for both pulse lengths used here. Note the length of the shorter pulse (0.5ms) represented here.

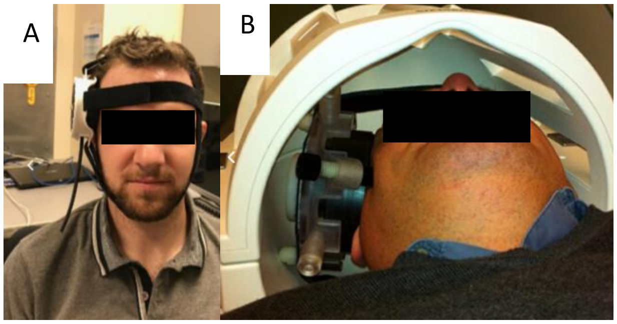

Figure S6: Ultrasound Stabilization. Example images of transducer strap system and transducer inside the head coil used here. Note that both images depict different models of transducers from the same manufacturer. The transducer used here was intermediate in size between these two devices. Note that, in this study, horizontal and

vertical straps were employed as in A. Moreover, in the current study, the transducer was stabilized using MRI wedge cushions (not shown) placed within the head coil. Unlike in B, we ensured minimal free space between the subject's head, the transducer, and the head coil by filling that space with cushions, ensuring minimal movement of the transducer due to subject head motion.

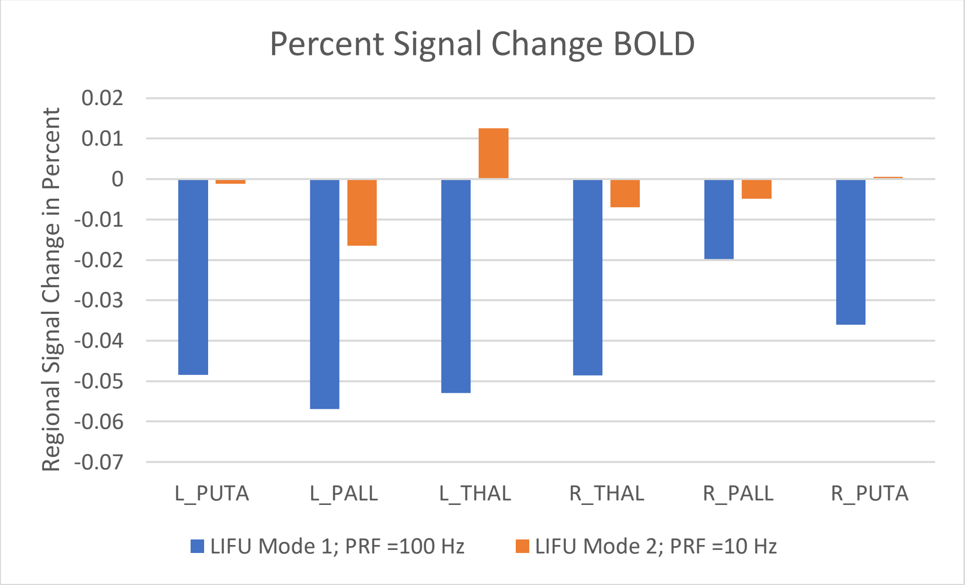

Figure S7: BOLD ROI Percent Change in Signal. Percent Change in Bold Signal for each ROI investigated in this study, segregated by Parameter set. These were extracted at the group level and so represent the mean and do not allow for the depiction of error bars. For assessment of statistical significance, see the main text.

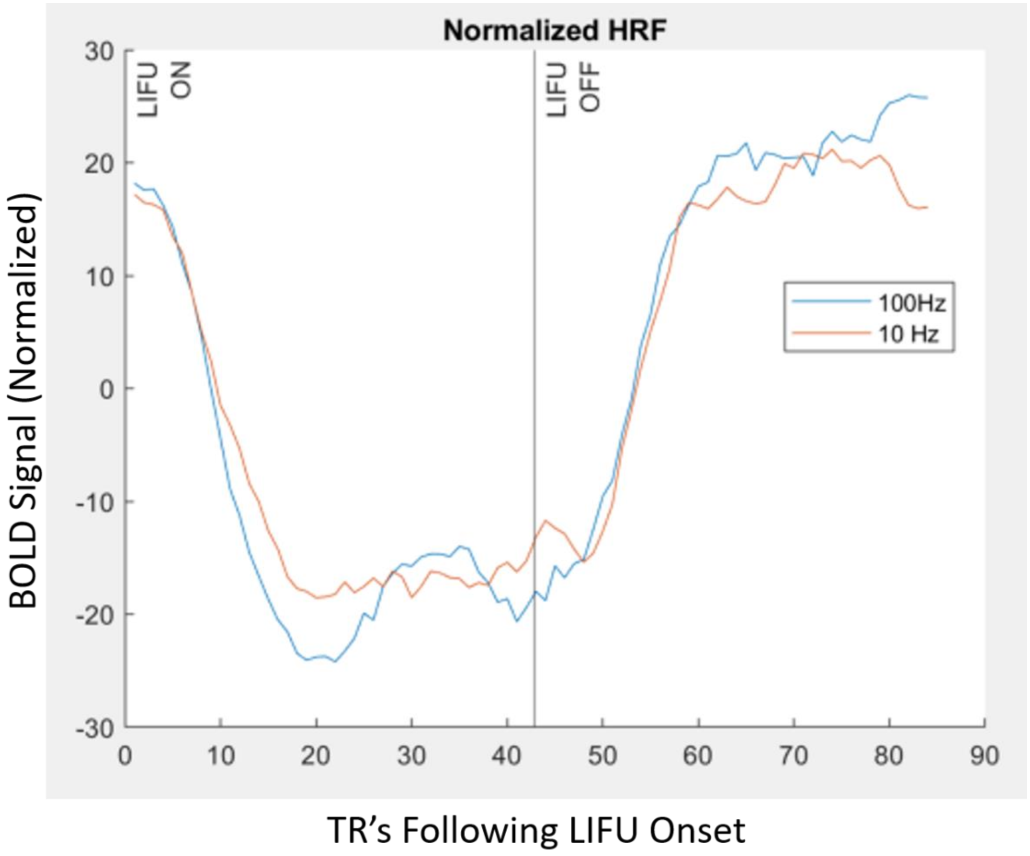

Figure S8: Study-Wide Averaged HRF. For both parameter sets, (Mode 1 (PRF = 100Hz); Mode 2 (PRF = 10Hz)), the average BOLD signal change is shown over the course of LIFU – on and LIFU – off blocks. Importantly, these plots are generated from regions that experienced inhibition exceeding a statistical threshold of $p > .005$ for each dataset and for each subject in order to capture the signal change within modulated regions. Thus, this data cannot be used to compare parameter sets or the degree of influence from LIFU, details addressed elsewhere in this report, but simply the shape of that influence. Qualitatively, the influence of LIFU appears to be generally sustained over the course of each block, as opposed to the possibility of a more short-lasting influence.

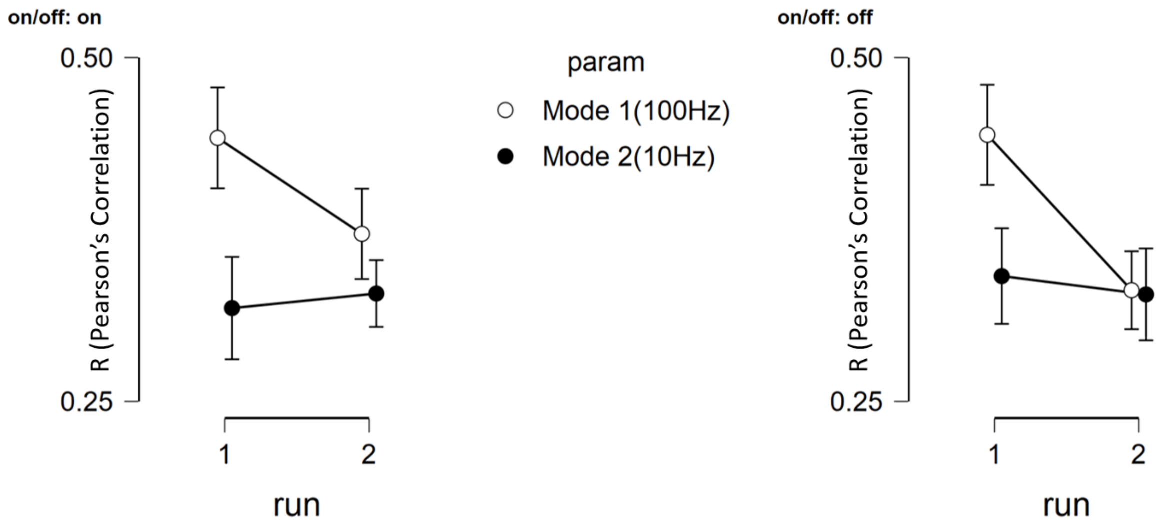

Figure S9: Pearson's Correlation between Left Globus Pallidus and Modulated Regions. Split by parameter set (mode 1 or mode 2), run (LIFU 1 or LIFU 2), and LIFU status (On or Off blocks), the correlation between the BOLD signal time series extracted from the left Globus pallidus and those regions for each subject which exceeded a $p < .005$ when modelling a decrease in BOLD signal during LIFU-on blocks. Only marginal effects were found when employing frequentist statistics, albeit with a high $BF_{inclusion}$ = 5.426 for the parameter variable, with Mode 1 apparently inducing more connectivity during run 1 when compared to mode 2 (see supplementary results). Error bars represent standard error.

Supplementary Results:

Follow-up Two Way ANOVAs (one per ROI extracted from ASL Data)

In the Left Putamen, a main effect of time point was found ($F_{Greenhouse\text{-}Geisser}$ (1.396, 20.102) = 4.873, p = .028; $BF_{Inclusion}$ = 0.824). Follow up revealed a more linear trend with no significant difference between Pre LIFU 1 and Post LIFU 1 t(15) = 2.537, p_{holm} = .074; BF_{10} = 0.654) or between Post LIFU 1 and Post LIFU 2, (t(15) = .840, p_{holm} = .407; BF_{10} = 1.325). In the Left GP, a main effect of time point was found ($F_{Greenhouse\text{-}Geisser}$ (1.141, 18.739) = 7.543, p = .011; $BF_{Inclusion}$ = 1.719). Follow up revealed a significant difference between Pre LIFU 1 and Post LIFU 1 was found 2 (t(15) = 2.867, p_{holm} = .015; BF_{10} = 1.159) with no difference between Post LIFU 1 and Post LIFU 2 found. In the Left Thalamus, a main effect of time point was found ($F_{Greenhouse\text{-}Geisser}$ (1.629, 21.512) = 7.816, p = .004; $BF_{Inclusion}$ = 3.707). Follow up revealed a significant difference between Pre LIFU 1 and Post LIFU 1 (t(15) = 3.561, p_{holm} = .004; BF_{10} = 8.242) with no difference between Post LIFU 1 and Post LIFU 1 found. In the Right Putamen, a main effect of time point was also found ($F_{Greenhouse\text{-}Geisser}$ (1.324, 19.862) = 4.604, p = .035; $BF_{Inclusion}$ = 0.497). Follow up revealed a significant difference between Pre LIFU 1 and Post LIFU 1(t(15) = 2.537, p_{holm} = .033; BF_{10} = 1.012). In the Right GP, a main effect of time point was found ($F_{Greenhouse\text{-}Geisser}$ (1.311, 19.662) = 4.547, p = .037 $BF_{Inclusion}$ = 0.504). Follow up revealed a more linear trend, a significant difference between Pre LIFU 1 and Post LIFU 2 (t(15) = 2.918, p_{holm} = .02; BF_{10} = 1.586). In the Right Thalamus, a main effect of Run was found ($F_{Greenhouse\text{-}Geisser}$ (1.760, 26.393) = 7.376, p = .004; $BF_{Inclusion}$ = 2.610). Follow up revealed a significant difference between LIFU 1 and 2 (t(15) = 3.609, p_{holm} = .003; BF_{10} = 7.388) with no difference between LIFU 2 and 3 found.

Pearson's Correlation Between Left GP and Activated Regions:

In order to assess if activated regions changed in their connectivity with the Left GP target during sonication, timeseries were extracted from BOLD data for regions that experienced apparent inhibition (decreased BOLD signal during LIFU-on compared to baseline) exceeding a statistical threshold of $p > 0.005$ for each first-level analysis as well as for the left Globus pallidus. The Pearson's correlation was calculated between these time series for LIFU-on and LIFU-off blocks for each parameter set and for each subject. A 2 x 2 x 3 repeated measures ANOVA (and it's Bayesian equivalent) was run with Parameter Set (Mode 1

or Mode 2), Run (Sonication 1 or Sonication 2 within session), and LIFU-status (“on” or “off”) as factors. No clear effects were found in the frequentist statistics; however, a marginal effect of run, $F(1,15) = 3.807$, $p = 0.07$, $BF_{inclusion} = 0.942$ and run by parameter interaction, $F(1,15) = 3.689$, $p = 0.074$, $BF_{inclusion} = 1.324$ were observed. Note that a high $BF_{inclusion} = 5.426$ was observed for the Parameter set variable in the repeated measures Bayesian ANOVA, forming a complex picture of these results which are dominated by a higher connectivity for both on and off blocks during Mode 1 for the first sonication (of two on the same day; see Figure S9).

Supplementary Methods:

Connectivity Psychophysiological Interaction (PPI):

Psychophysiological analysis was conducted to determine which, if any, regions of the whole brain changed their connectivity with the left globus pallidus during 30s blocks of sonication as compared to non-sonication blocks. In essence, the "psychological" factor here is the time course of LIFU, instead of the time course of a subject-driven action, as is more typical. The same masks used for the BOLD ROI analyses (see Block Design BOLD ROI) were used here. This was done for data from each BOLD sequence (4 per subject) using FSL's tool for extracting the time points of selected voxels in a 4D image (fslmeants)[1]. Using FSL FEAT, each LIFU-BOLD data sequence was first analyzed employing a multivariate general linear model (GLM) approach including our block design, the time series of the ROI, and importantly, their interaction. Finally, lower-level results from each LIFU mode were aggregated respectively using a mixed effects FLAME 1 + 2 model. Aggregated data were regressed on subject of origin. Data were cluster-corrected for multiple comparisons using a cluster-level threshold of $z > 3.09$ (corrected $p < 0.05$), as well as, in a separate third level analysis, $z > 2.57$ (corrected $p < 0.05$). This analysis returned no significant results and so no results are depicted for it.

Study-Wide Averaged HRF:

For each BOLD dataset and for each subject, time series were extracted for regions where BOLD signal decreased during sonication (as compared to baseline) as apparent inhibition was the only effect found at the group level. These regions were selected on a per-subject basis by thresholding statistical images at a statistical threshold of $p > .005$ at the first level analysis and binarizing them to produce masks. The time series of these regions were then extracted using the "fslmeants"[1] command with the thesholded masks. In Matlab (v.2020a) , these time series were segmented by the LIFU-design such that 10 roughly (some variability between subjects) 60 second chunks were created from each time series beginning at the first moment of each 30s LIFU administration and ending at the end of the 30s LIFU – off period. Blocks were aggregated within and then between subjects for each parameter set.

Pearson's Correlation:

Time series for the Left Globus Pallidus and for the modulated regions (see Methods for "Connectivity Psychophysiological Interaction (PPI)" and "Study-Wide Averaged HRF" for extraction procedure) were assessed for

the degree of dependency (Pearson's Correlation) in Matlab (v.2020a) using the "corrcoef" function. Pearson's Correlation was calculated for periods of LIFU-on and periods of LIFU-off (baseline) for each run (Sonication 1 or Sonication 2 within session), for each parameter set, and for each subject. These values were exported to Jasp (Version 0.11.1) for statistical analysis. A 2 x 2 x 3 repeated measures ANOVA (and its Bayesian equivalent) was run with Parameter Set (Mode 1 or Mode 2), Run (Sonication 1 or Sonication 2 within session), and LIFU-status ("on" or "off") as factors.

References

1. Jenkinson, M., Beckmann, C. F., Behrens, T. E. J., Woolrich, M. W. & Smith, S. M. FSL. *NeuroImage* **62**, 782–790 (2012).
